# A Risk-Based Clinical Decision Support System for Patient-Specific Antimicrobial Therapy (iBiogram): Design and Retrospective Analysis

**DOI:** 10.2196/23571

**Published:** 2021-12-03

**Authors:** Lars Müller, Aditya Srinivasan, Shira R Abeles, Amutha Rajagopal, Francesca J Torriani, Eliah Aronoff-Spencer

**Affiliations:** 1 Design Lab University of California San Diego La Jolla, CA United States; 2 Division of Infectious Diseases and Global Public Health Department of Medicine UC San Diego Health La Jolla, CA United States

**Keywords:** antimicrobial resistance, clinical decision support, antibiotic stewardship, data visualization

## Abstract

**Background:**

There is a pressing need for digital tools that can leverage big data to help clinicians select effective antibiotic treatments in the absence of timely susceptibility data. Clinical presentation and local epidemiology can inform therapy selection to balance the risk of antimicrobial resistance and patient risk. However, data and clinical expertise must be appropriately integrated into clinical workflows.

**Objective:**

The aim of this study is to leverage available data in electronic health records, to develop a data-driven, user-centered, clinical decision support system to navigate patient safety and population health.

**Methods:**

We analyzed 5 years of susceptibility testing (1,078,510 isolates) and patient data (30,761 patients) across a large academic medical center. After curating the data according to the Clinical and Laboratory Standards Institute guidelines, we analyzed and visualized the impact of risk factors on clinical outcomes. On the basis of this data-driven understanding, we developed a probabilistic algorithm that maps these data to individual cases and implemented iBiogram, a prototype digital empiric antimicrobial clinical decision support system, which we evaluated against actual prescribing outcomes.

**Results:**

We determined patient-specific factors across syndromes and contexts and identified relevant local patterns of antimicrobial resistance by clinical syndrome. Mortality and length of stay differed significantly depending on these factors and could be used to generate heuristic targets for an acceptable risk of underprescription. Combined with the developed *remaining risk* algorithm, these factors can be used to inform clinicians’ reasoning. A retrospective comparison of the iBiogram-suggested therapies versus the actual prescription by physicians showed similar performance for low-risk diseases such as urinary tract infections, whereas iBiogram recognized risk and recommended more appropriate coverage in high mortality conditions such as sepsis.

**Conclusions:**

The application of such data-driven, patient-centered tools may guide empirical prescription for clinicians to balance morbidity and mortality with antimicrobial stewardship.

## Introduction

### Background

Antibiotic-resistant infections are widespread in the United States and across the globe and are an urgent threat to human health [1,2]. In the United States, over 2.8 million resistant infections occur each year, leading to delayed or ineffective therapy, longer hospital stays, and increased risk of death [1]. Patients who receive early empiric therapy that matches the in vitro susceptibility of their infection are up to 12 times more likely to survive (30-day crude mortality) [3], have shorter length of stays (LOSs) [4], and less long-term sequelae [5]. At the same time, overuse and prescription of overly broad antibiotics increases the risk of antimicrobial resistance (AMR), creating a positive feedback loop between behavior and ecological response, perpetuating a vexing health dilemma that spans sociobehavioral, ecological, and technical dimensions [6]. Increased antibiotic use in response to the COVID-19 pandemic exemplifies this complexity with the potential to further amplify the risk of AMR [7,8].

To combat the global AMR crisis today, leading agencies (eg, World Health Organization, Infectious Disease Society of America, Centers for Disease Control and Prevention, Agency for Healthcare Research and Quality, and the Clinical and Laboratory Standards Institute) have recommended that health care institutions produce and distribute locally derived antibiograms to clinicians, primarily as part of antimicrobial stewardship efforts [9,10]. Antibiograms have traditionally been high-level printed tabular summaries of local resistance patterns within an institution. These tables do not account for many factors known to influence antimicrobial susceptibility [11,12], even though such data are available in the electronic health record (EHR).

Recent machine learning reports demonstrated that EHR data can predict susceptibility, although generally for single disease types [13-15]. For instance, Yelin et al [13] used a data set of over 700,000 community-onset urinary tract infections (UTIs) to show that the use of prior antibiotics predicts AMR. The underlying machine learning techniques require large data sets and are therefore best suited for common infections, such as uncomplicated UTIs [13,15]. Different approaches are needed for smaller data sets, for example, to understand the influence of rare patient-specific factors or local susceptibility patterns.

Few hospitals have incorporated patient-specific and epidemiologic information into traditional antibiograms [16] or clinical decision support systems (CDSSs) [17-19]. Unit-specific or syndrome-specific antibiograms have been developed [20-22]. Visual analytics software have been studied as a tool for integrating data within an EHR on patient risk factors with isolate-specific susceptibilities [23]. The TREAT system is one of the most comprehensive and well-documented diagnostic and antimicrobial decision support tools [24,25]. TREAT uses a sophisticated causal probabilistic network [24] that hybridizes expert knowledge with heuristics and local antibiotic susceptibility data. In a multinational randomized controlled trial in 2013, TREAT use for inpatients led to a decreased LOS (1 day) but did not show mortality benefits when all variables were accounted for [25].

Reviews of clinical decision systems for antimicrobial management [17-19] found a paucity of evidence that these systems reduce mortality and morbidity when incorporated into daily clinical workflow, an unnatural segregation of outpatient and inpatient approaches, and a lack of systematic engagement with stakeholders about needs and workflow integration for support systems. A recent survey showed that only 44% of medical residents knew how to access the local antibiogram and preferred web-based resources such as UpToDate or the Sanford Guide [26]. These web-based treatment guidelines are not tailored to a specific health care environment as local antibiograms, but they are more accessible and provide explicit guidance for a case at hand. Thus, decades after the first digital innovations, the traditional antibiogram, with all its limitations, is still the most common AMR tool in use today.

### Objectives

In this study, we introduce iBiogram as a digital CDSS for data-driven antimicrobial selection in the absence of definitive antibiotic susceptibilities. iBiogram addresses the limitations discussed above by examining infectious disease decision support as a complex sociotechnical problem [6,27]. Physicians and CDSS have to work together to complement the understanding of a specific case with guidance about the local epidemiology, likelihood of resistance, and associated risk of failure. Shared representations between algorithms and people are the key to blending the best qualities of expert knowledge and digital tools for efficient human technology teamwork in such cases [28]. Shared representations establish a common language to translate between clinical expertise and machine inference to enable efficient human-machine collaboration. To this end, we explored diverse patient information and antimicrobial testing results, presented new visualizations of insights generated from these data, and share the results of evaluating a new prototype and risk-based metric compared with historic provider behavior.

## Methods

### Overview

We systematically evaluated a comprehensive clinical data set that crossed ambulatory and hospital settings and contained antimicrobial testing, clinical context, and patient factors. We then identified local factors influencing microbial prevalence and AMR, their impact on mortality, and how they combine to influence the success of antibiotic treatments. Finally, we developed and tested a decision support tool that may be used in both low-risk ambulatory settings where overprescriptions likely dominate and inpatient settings where the risks and benefits of early and accurate empiric therapy are the greatest.

### Data Source and Analysis

We analyzed 5 years of antibiotic susceptibility isolate testing data and related deidentified patient information from the University of California San Diego Health Sciences (UCSDHS). The study was reviewed and approved by the University of California San Diego Human Research Protections Program (Institution Review Board #161853). Positive bacterial cultures with susceptibility results between May 2011 and November 2016 were included. Contents included identified pathogens; their susceptibility to tested antibiotics; diagnostic information from before, during, and after the ordering of cultures; any medications prescribed before and throughout their visit; the problem list at the time of order; and general demographic information.

We processed the data before analysis by removing repeat susceptibility tests according to the Clinical and Laboratory Standards Institute guidelines [9]. Suppressed antibiotics and supplemental tests performed after cultures returned were not considered for the purposes of this study. The International Classification of Diseases-10 (ICD-10) codes at order and at discharge were mapped to syndromes and prevalent comorbidities; for example, sepsis was defined as a positive blood culture and a corresponding ICD-10 code. An overview of the mapping is provided in Multimedia Appendix 1.

We studied the influence of time, demographics, assigned syndromes, medications, and comorbidities on the resulting distribution of causal bacteria, their susceptibility, and clinical outcomes. Logistic regression was used to calculate the odds ratios (OR) adjusted for age and sex, and the results were filtered by significance (P<.05). The influence of ineffective therapy on clinical outcomes was assessed between syndrome-factor combinations. Critical combinations were identified by evaluating changes in 7-day all-cause mortality and median LOS for prescribed treatments.

The impact of factors and comorbidities on the resulting distribution of causal bacteria, their susceptibility, and mortality were analyzed using the Python statsmodels package implementation of logistic regression adjusted for age and sex to account for significant differences in syndrome types and commonly occurring organisms, for example, higher rates of UTI and gram-negative bacteria in women. Age was modeled as a categorical variable (0-14, 15-24, 25-44, 45-64, and ≥65). The impact of age and sex was evaluated by controlling for each other. The change in LOS was analyzed using the Mann-Whitney *U* test from the scipy package. The effect size in days was calculated as the difference in medians to account for skewed distribution.

### Algorithm and iBiogram Decision Support Platform Development

To analyze and communicate the impact of risk factors for specific cases, we developed the remaining risk metric as a shared representation [28]. The risk of ineffective empiric antibiotic therapy was calculated as the sum of the individual resistance probabilities of each possible causal agent *r_i_* weighted by the probability of that causal agent *a_i_*. With n referring to the number of possible causal agents:



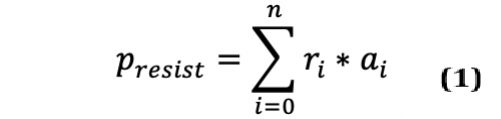



The remaining risk of combination therapy was calculated by selecting the minimal *r_i_* of all therapy antibiotics. The result was a prevalence-weighted multiantibiotic antibiogram that we refer to as iBiogram. We then developed a web-based prototype of the iBiogram where all statistically significant factors may be coselected to generate a patient-centric antibiogram, as well as a recommended set of antibiotics with the best coverage for predicted organisms.

The iBiogram algorithm draws antibiotics from a knowledge base that maps antibiotics to syndromes. Antibiotics are additionally filtered by the available test data for the selected scenario to prevent inflated rankings caused by a few or, in the worst case, a single antibiotic susceptibility isolate test. All possible antibiotic combinations were generated and ranked based on the remaining subset of antibiotics. Sepsis is currently the only syndrome that allows the combination of three antibiotics with amikacin and gentamicin reserved as the third drug.

### Evaluation

The iBiogram algorithm was evaluated by using data before the last 6 months and comparing the performance of predicted *best* treatments to the actual treatments and outcomes in the last 6 months of the data. Physicians’ decisions and iBiogram prediction decisions were evaluated as successful if the identified organism was susceptible to any of the prescribed or recommended antibiotics. For physicians’ empiric therapy choices, we considered antibiotics prescribed between 24 hours before and 8 hours after the culture was ordered. Considering antibiotic stewardship and building on the conducted mortality analysis, we used predicted-susceptibility targets developed by Cressman et al [29] to rank potential treatments for a given syndrome based on the risk of mortality or prolonged LOS. Prescribed and suggested therapeutic regimens were further scored on the use of protected antibiotics and essential medicines according to World Health Organization’s Action for Welfare and Awakening in Rural Environment Classification Database [30]. This classification groups therapies into *Access group Antibiotics*, those that are broadly active but minimal risk for resistance, *Watch group antibiotics* that are high priority therapies at substantial risk for developing resistance without stewardship, and *Reserve group antibiotics* that are saved only for use in life-threatening multidrug-resistant infections.

## Results

### Data Summary

**Figure 1:**
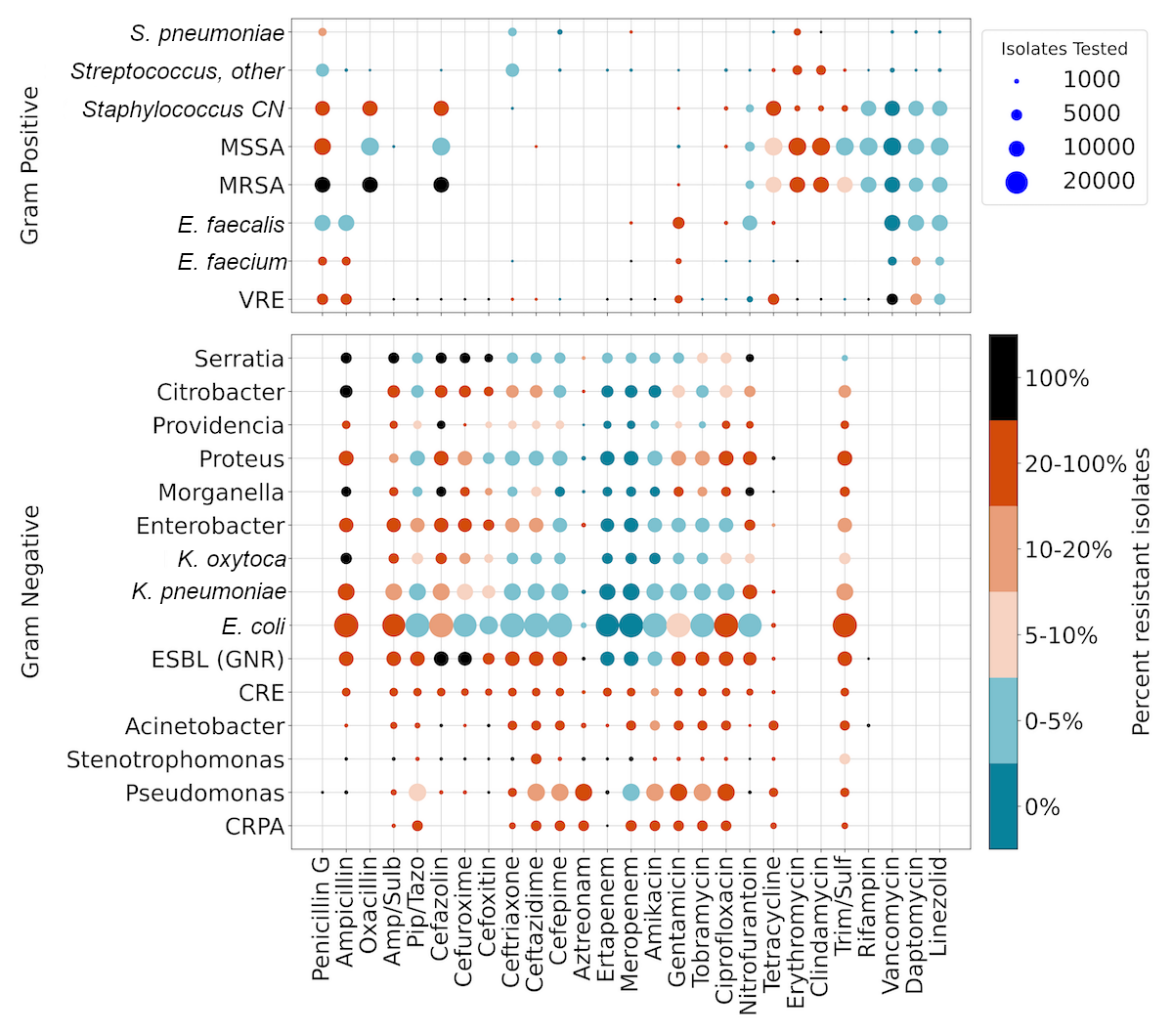
Overview of antibiotic resistance: Summary of the analyzed data for the most commonly tested antibiotics and causal agents. Each dot represents all isolates with a given causal agent (horizontal) and tested antibiotic (vertical). The number of isolates is indicated by dot-size and the probability of resistance is encoded as color ranging from blue (0% resistance) over red (>20% resistance) to black (100% resistance). CN: Coagulase Negative Staphylococci; CRE: Carbapenem-resistant enterobacteriaceae; CRPA: Carbapenem-resistant Pseudomonas aeruginosa; ESBL: Extended Spectrum Beta Lactamase producing Enterobacteriaceae; GNR: Gram negative rod; MRSA: Methicillin-resistant Staphylococcus aureus; MSSA: Methicillin-susceptible Staphylococcus aureus; VRE: Vancomycin-resistant Enterococcus.

Figure 1 depicts an overview of all susceptibility tests for gram-positive and gram-negative organisms over the full 5-year data set at the UCSDHS. A complete table is available in the Multimedia Appendix 2. Here, we re-envision a general static antibiogram, summarizing prevalence (dot area) and resistance patterns (red is increasing prevalence). Although printed antibiograms display only the current quarter, including more data can result in a higher predictive value for a specified patient, as a larger number of cases reduces the effect of outliers. An analysis of larger time windows found no significant differences between quarterly antibiograms and data, including up to 7 quarters (Multimedia Appendix 3).

### Factors Influencing Microbial Ecology, Resistance, Mortality, and LOS

**Figure 2:**
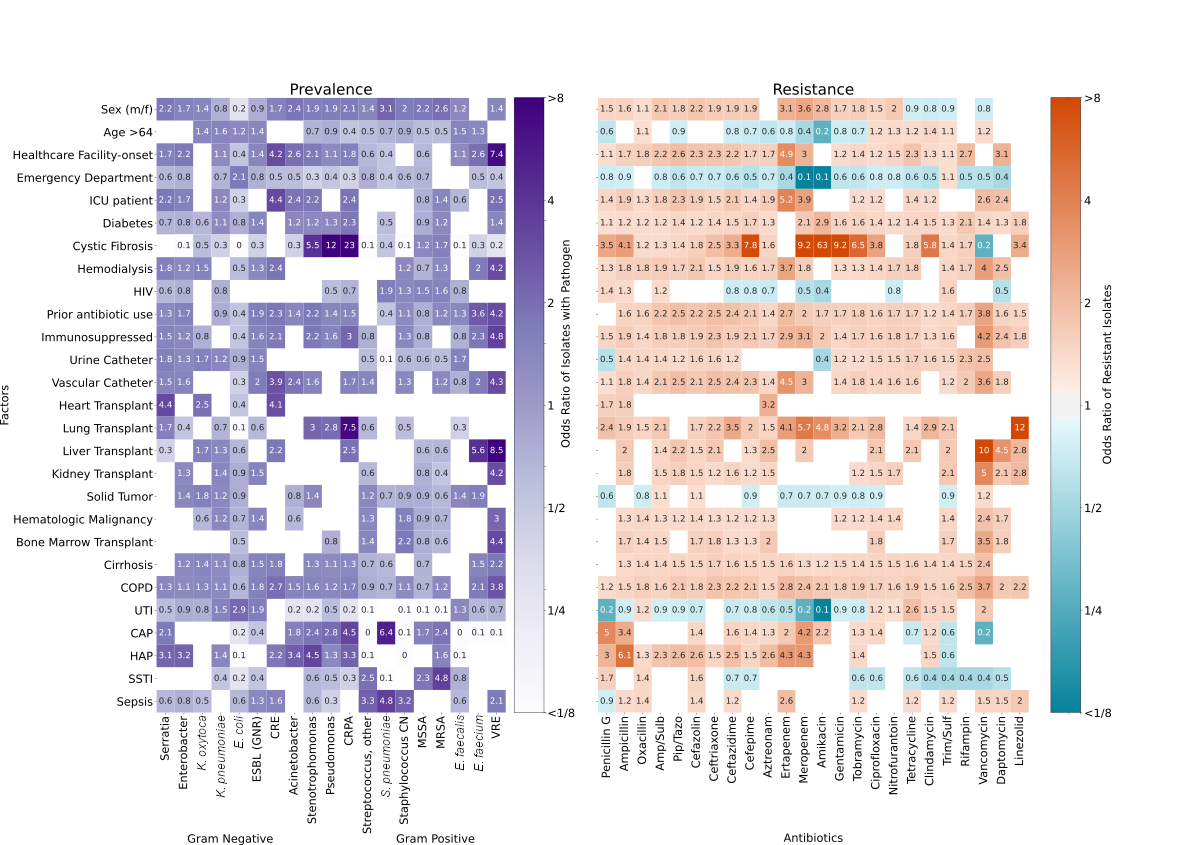
Factors influencing microbial prevalence (left) and resistance (right): Odds ratios for statistically significant factors (*P*<.05) influencing the probability of encountering a specific bacterium or an isolate resistant to a particular treatment were adjusted by sex and age. Sex and age were adjusted against each other. Factors include demographic information, zip code, medications, syndromes, and comorbidities as coded in International Classification of Diseases-10. In the left graph, purple indicates a higher prevalence of particular pathogens. In the right graph, red indicates a higher and blue a lower prevalence of resistant isolates. CAP: community-acquired pneumonia; CN: coagulase-negative Staphylococci; COPD: chronic obstructive pulmonary disease; CRE: carbapenem-resistant Enterobacteriaceae; CRPA: carbapenem-resistant Pseudomonas aeruginosa; ESBL: extended-spectrum β-lactamases; GNR: Gram negative rod; HAP: health care–associated pneumonia; ICU: intensive care unit; MRSA: methicillin-resistant Staphylococcus aureus; MSSA: methicillin-susceptible Staphylococcus aureus; SSTI: skin and soft tissue infection; UTI: urinary tract infection; VRE: vancomycin-resistant Enterococcus.

The risk factors influencing organism prevalence and resistance at UCSDHS range from well-known contributing factors such as prior antibiotic use to more specific insights, such as the influence of a specific transplant history. Figure 2 depicts the principal factors that drive AMR, Multimedia Appendices 3-6 supply additional data. They were assessed and reported as adjusted ORs for changes in organism prevalence (left) and resistance (right). Expected contributing factors included sex, age, and context, such as whether a patient presented in an outpatient setting, to an emergency department, or developed illness while hospitalized. Certain historic patient factors (eg, diabetes, cystic fibrosis, hemodialysis, or history of transplantation) were linked to significantly higher rates of multidrug-resistant organisms. For instance, patients on hemodialysis had higher rates of carbapenem-resistant Enterobacteriaceae (CRE; OR 2.4, 95% CI 1.7-3.3). Patients with health care–associated pneumonia were at increased risk for *Acinetobacter* (OR 3.4, 95% CI 2.7-4.4), CRE (OR 3.3, 95% CI 2.7-4.1), and carbapenem-resistant *Pseudomonas aeruginosa* (CRPA; OR 3.3, 95% CI 2.7-4.1). Cystic fibrosis presented the strongest effects in both ecology and resistance, showing drastic shifts to *Pseudomonas* (OR 12.4, 95% CI 11.4-13.5), CRPA (OR 22.7, 95% CI 18.8-27.3), *Stenotrophomonas* (OR 5.5, 95% CI 4.5-6.7), and methicillin-resistant *Staphylococcus aureus* (OR 1.7, 95% CI 1.5-1.9), with significant decreases in other pathogenic organisms.

**Figure 3:**
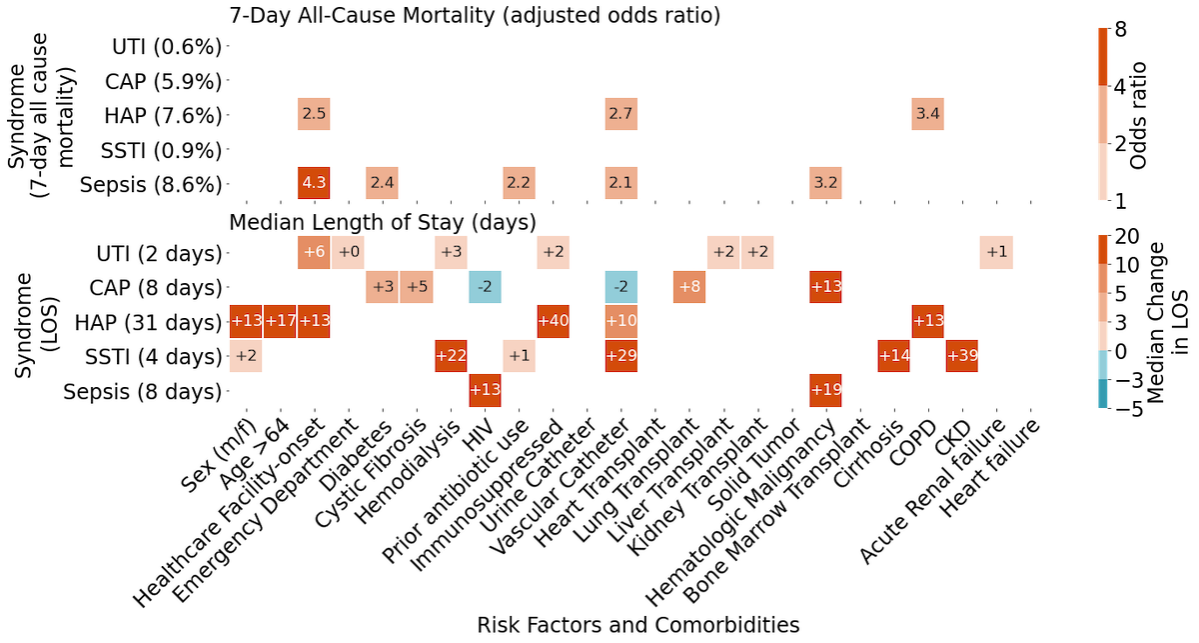
Influence of treatment failure on 7-day all-cause mortality (top) and median length of stay (bottom) in syndromes/risk factor combinations: A summary of the influence of resistant pathogens for syndromes modified by risk factor or comorbidity. Only significant results (*P*<.05) are shown. The top graph shows the age and sex adjusted 7-day all-cause mortality odds ratio for failed treatments, for example, hospital-facility sepsis patient with ineffective treatment are 4.3 times more likely to die within 7 days. The bottom graph depicts how ineffective treatment affects the median length of stay for the same combinations including only surviving patients and testing significance using the Mann-Whitney *U* test. For each syndrome, the mean mortality and the median length of stay is listed on the y-axis behind the syndrome name in brackets. CAP: community-acquired pneumonia; CKD: chronic kidney disease; COPD: chronic obstructive pulmonary disease; HAP: health care–associated pneumonia; SSTI: skin and soft tissue infection; LOS: length of stay; UTI: urinary tract infection.

The syndrome-associated 7-day all-cause mortality, as well as median LOS, differed significantly based on sensitivity to empiric treatments. The specific syndrome factors are reported in Figure 3. Failure to prescribe active antibiotics for patients presenting with a UTI or community-acquired pneumonia did not significantly influence mortality or resulted in small changes in the LOS, whereas cases of health care–associated pneumonia and sepsis showed significant increases in mortality and LOS. For instance, nosocomial sepsis with hematologic malignancy was significantly associated with increased 7-day all-cause mortality (OR 3.2) and LOS (+19 days) when empiric antibiotics were inactive against the causative pathogen.

### Visualizing Risk of Resistance for Empiric Therapy

**Figure 4:**
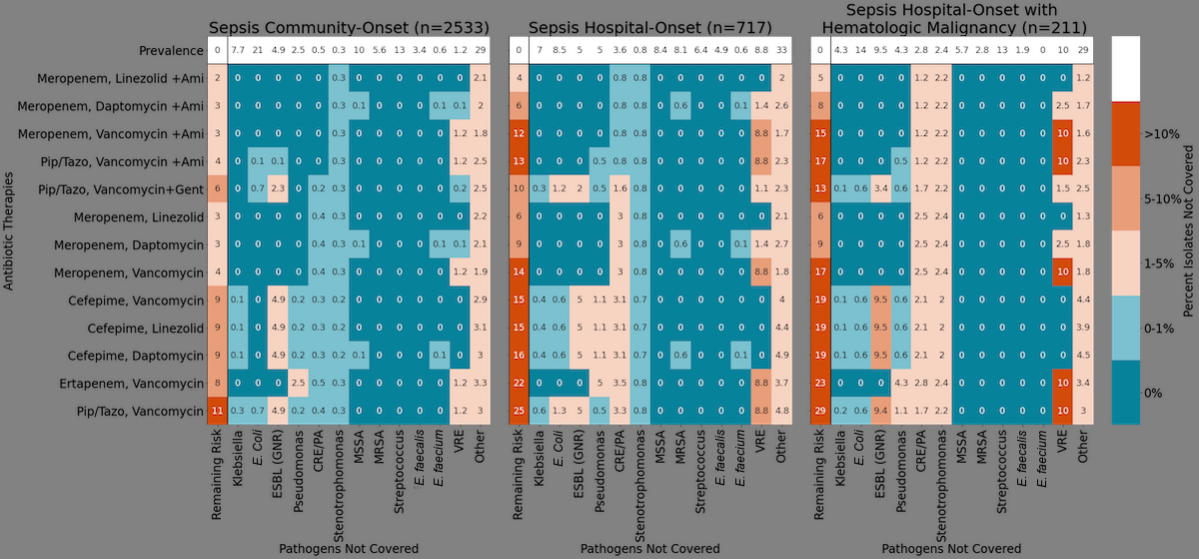
Remaining risk of treatment failure in sepsis: A summary of the percent risk of failure for common multidrug regimens in sepsis cases. Shown are blood culture summations from community-onset sepsis, hospital-onset sepsis, and hospital-onset sepsis in patients with a hematologic malignancy. Numeric values represent remaining risk (prevalence x predicted resistance) to a given antibiotic or antibiotics. The prevalence (top row) lists the probability of encountering the delineated causal agents. If a treatment does not cover a pathogen the risk equals the prevalence of that pathogen, for example, meropenem never covers methicillin-resistant Staphylococcus aureus. The percentage in the remaining risk column (leftmost) sums the probability of encountering a pathogen not covered by the delineated therapy. Blue color coding represents <1%, increasing in red intensity until, red ≥10%, a minimum threshold suggested for severe infection [29]. CRE: carbapenem-resistant Enterobacterales; ESBL: extended-spectrum β-lactamases; GNR: Gram negative rod; MRSA: methicillin-resistant Staphylococcus aureus; MSSA: methicillin-susceptible Staphylococcus aureus; PA: Pseudomonas aeruginosa; VRE: vancomycin-resistant Enterococcus.

The difficult tradeoff between overly broad empiric regimens and the risk of treatment failure requires a shared representation that accounts for known risk factors. Consider the scenario shown in Figure 4 where on the left, we show a filtered remaining risk antibiogram displaying the performance (chance of failure) of common empiric antibiotic regimens for patients presenting with community-onset sepsis in the emergency department. In the middle, we consider changes in risk associated with hospital-onset sepsis and on the right for the subset of these patients with a hematologic malignancy.

An antibiogram for community-onset sepsis would predict the frequently prescribed empiric therapy of vancomycin and piperacillin/tazobactam to fail in 11% of pathogens in this data set. However, after adding hospital-onset and hematologic malignancy factors, the failure risk increases to 25% and over 28%, respectively, each below the recommended target of complete or at a minimum 90% coverage (<10% remaining risk) in cases of severe sepsis [29]. At first glance, daptomycin and meropenem, less traditional first-line agents for hospital-onset sepsis, might be considered a better empiric regimen in these populations, with only 9% remaining risk of failure.

### Envisioning a Human-Centered Antibiogram

**Figure 5:**
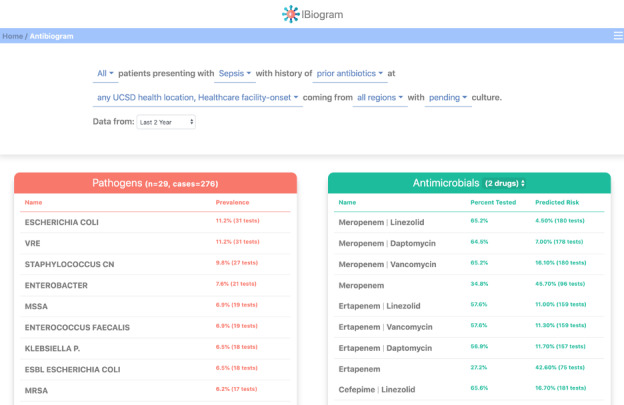
The iBiogram digital antibiogram: iBiogram is a web-based app that allows the user to select patient demographics, presenting syndrome, prior use of antibiotics or immune suppression, comorbid conditions, as well as the location and context of infection, the region a patient has come from and if the pending cultures have been identified as gram-positive or -negative. In the example above, all patients with diabetes on prior antibiotics and presenting with nosocomial sepsis over 2 years are shown. If the user suspects a specific organism based on history that organism can be selected, and the table will reorient to show the sensitivities for just that organism. Likewise, a user can select a treatment regimen and examine the organisms that are sensitive or resistant to the treatment. ESBL: extended-spectrum β-lactamases MRSA: methicillin-resistant Staphylococcus aureus; MSSA: methicillin-sensitive susceptible Staphylococcus aureus; VRE: vancomycin-resistant Enterococcus.

In Figure 5, we provide a prototype digital antibiogram capable of accounting for the factors that predict the particular ecology associated with patients sharing these factors, along with the associated resistance patterns and suggested antibiotics with their expected coverage. Here, we selected all patients over the last eight quarters who developed nosocomial sepsis while receiving antibiotic therapy. In the left panel, we can see the most prevalent pathogens in this population. The user can scroll down to see the long tail of the organisms. On the right, a list of potential treatments (1-3 drugs may be displayed) is shown with predicted coverage, and the ability to toggle and show the remaining risk. Overall, using a combination of patient factors, historic culture data, and antibiotic prescribing rules, the user can readily select key factors to generate a patient-level antibiogram that is tailored to the case at hand. The system then provides a list of therapeutic options (ranked either by predicted coverage or by remaining risk) and suggests treatments that a provider may select based on further details such as patient history of medication allergies, need for bactericidal versus bacteriostatic drugs, preferred route of administration, drug-drug interactions, and cost of treatment.

### Comparing Empiric Therapy at the Time of Order Versus iBiogram Suggestions

Given that patients who received effective empiric therapy were more likely to survive and had shorter LOS, we analyzed the failure rate of prescribed empiric therapies based on final sensitivity results and compared them to the suggested iBiogram regimens (Table 1). For patients presenting to the emergency department with a UTI, the most commonly prescribed antibiotics were ciprofloxacin and ceftriaxone, and the overall failure rate was 17.7% (85/479), whereas the suggested iBiogram treatments would have been between 23% and 15.1%. For the subset of patients with UTIs in the emergency department with prior antibiotics, the empiric therapy treats only 74% (48/65; 26.2% failure), whereas suggested regimens would have achieved a reduction of 3.6%-14.2%. For community-onset sepsis, the most frequently prescribed drugs were piperacillin/tazobactam and vancomycin, and the overall failure rate considering all cases was 8.1% (31/383), compared with 5.5% for the iBiogram-suggested treatment. For cases of hospital-onset sepsis, however, where there is significant associated mortality, prescribed empiric failure rose to 16% (12/75) in comparison to 7.8% using the proposed antibiotics. Finally, in cases of hospital-onset sepsis in patients with hematologic malignancy, where we found the strongest correlation of mortality and LOS with sensitivity to prescribed antibiotics, we see failure of empiric therapy climb to 17.4% (4/27) failure, whereas suggested combinations would have failed in only approximately 13% of cases.

**Table 1:** Comparison of the performance of iBiogram recommendations to actual antibiotics prescribed by doctors at the time of order.^a^

Syndrome	Risk factor			Received empiric antibiotics			Retrospective comparison of prescribed empiric therapies to iBiogram suggestions					
	Total	Deceased, n (%)	Total		Deceased, n (%)	Empiric therapy (%)						iBiogram suggestion
						Measured risk		AWaRe^b^ group access	AWaRe group watch	AwaRe group reserve	Measured risk (%); predicted risk; AWaRe group	
UTI^c^ in the ED^d^	939	0 (0)	479		0 (0)	17.7		24	76	0	23%, nitrofurantoin; 19.4%; access 15.1% ceftriaxone; 17.8%; watch	
UTI in the ED on antibiotics	147	0 (0)	65		0 (0)	26.2		26	74	0	1.9%, amikacin; 19.3%; access 18.7% nitrofurantoin and trimethoprim/sulfamethoxazole; 19.9%; access	
Sepsis, community onset	562	43 (7.7)	383		30 (7.8)	8.1		6	92	2	14.2%, vancomycin and piperacillin/tazobactam; 9.8%; watch 5.5% vancomycin and ertapenem; 8.2%; watch	
Sepsis, hospital onset	181	17 (9.4)	75		7 (9.3)	16		1	95	4	7.8% daptomycin and meropenem; 7.6%; reserve 3.6% daptomycin and meropenem+amikacin; 6.8%; reserve	
Sepsis, hospital onset with hematologic malignancy	39	8 (20.5)	23		4 (17.4)	17.4		4	91	4	15.5% daptomycin and meropenem; 7.4%; reserve 12.9% daptomycin and meropenem+gentamicin; 5.1%; reserve	

^a^Compares observed prescription and success rates for prescribers in the last 6 months of the study versus suggested iBiogram regimens for cases with prescriptions based on all prior data. Noted mortality rates are a proxy for risk of using ineffective antimicrobial therapy.

^b^AWaRe: Action for Welfare and Awakening in Rural Environment.

^c^UTI: urinary tract infection.

^d^ED: emergency department.

## Discussion

### Research in Context

On the basis of gaps in the science noted by Curtis [17], Rawson [18], and Laka [19], this study adopted a systematic human-centered approach to analyze, visualize, and operationalize factors associated with AMR, mortality, and LOS of bacterial infections across a United States academic health care system, spanning from ambulatory and emergency to inpatient and intensive care settings. Our efforts differed from machine learning approaches that remain limited to large homogenous data sets, as well as from the robust TREAT program [25], which incorporates more assumed knowledge but may miss factor associations. This study mirrors significant efforts such as Detecting and Eliminating Bacteria Using Information Technologies (DeBUGIT) [31], Wise Antimicrobial Stewardship Support System (WASPSS) [32], and EPiC IMPOC (Enhanced, Personalized and Integrated Care for Infection Management at the Point-Of-Care) [33,34] that have each sought to optimize bug-drug combinations by fusing data-driven approaches with expert consensus or guidelines. However, we propose that our integration of morbidity and mortality with clinical and microbiological data to present the risk of failure in a mortality cost-benefit context may be more intuitive to clinicians balancing patient safety and antimicrobial stewardship across the continuum of care.

We conducted a comprehensive examination of the factors influencing microbial ecology and resistance within a large urban and suburban health care system to set the stage for the development of an antibiotic recommendation system that can support clinicians prescribing antibiotics in the absence of culture data. We identified predictive factors that may be used to create tailored empiric antibiotic choices; however, these factors may be difficult to convey using static and nondigital approaches. Alone, these factors are also insufficient to make optimal therapeutic decisions, as recommendations to prescribers require fusion of purely probabilistic approaches with clinical heuristics, expert guidelines, and other risk benefit considerations [24].

We cleaned and created a new graphical representation of the bacterial prevalence and antibiotic resistance data. At a glance, this approach can be seen to be more readily informative of both the likelihood of encountering given pathogens and their expected pattern of resistance than current paper-based antibiograms. This approach also demonstrates the limitations of a hospital-wide and static view of AMR when attempting to map data to a specific patient and immediately suggests that more granular patient-specific data could inform better decision-making. Such a graphical overview could be generated for subsets of the data to generate and track a *fingerprint* of resistance patterns.

The complex sepsis cases in Figure 4 highlight the tension between the need for more precise, patient-specific, and readily accessible antibiograms but also indicate that actionable support for clinicians requires a complex understanding of patient factors and best practices that may be specific to an institution. For instance, closer examination and expert clinical experience revealed that daptomycin may offer little advantage over vancomycin against methicillin-resistant *Staphylococcus aureus*, merely adding coverage against vancomycin-resistant enterococci, which is generally encountered in specific situations and may be associated with higher mortality if missed empirically in these cases [35]. Likewise, the benefits of using a third agent such as amikacin, a potentially ototoxic and nephrotoxic drug, over the safer meropenem and daptomycin combination, would only be worthwhile in specific risk groups with higher rates of CRE and CRPA, such as patients with cystic fibrosis or a history of lung transplantation. The robust performance of amikacin in our data set also highlights the potential influence of a hospital’s formulary, as amikacin is rarely used and is not routinely stocked in the study hospital, where other aminoglycosides are favored. The ability to track antibiotic use with antibiotic resistance data on broad scales and among various institutions with different prescribing practices will ultimately shed important light on antimicrobial stewardship priorities.

To this end, we developed a digital tool combining up-to-date hospital data with clinical rules (here syndrome-specific antibiotics from expert guidelines) as an interactive precision tool for the selection of antibiotic combinations. This tool covers many typical scenarios encountered by clinicians across the continuum of care, ranging from empiric treatment of a UTI in ambulatory patients to solid-organ transplant recipients with sepsis presenting to the emergency room and the care of complex hospitalized patients such as those with stem cell transplants or cystic fibrosis. Notably, this ecology is highly regional and often hospital specific, highlighting the need to incorporate local organism prevalence, local formulary, and patient-specific risk factors. In the future, the tool could be readily integrated into EHRs to alert clinicians when prescribed regimens are too broad in coverage or have higher than accepted chances of failure. Alternatively, more appropriate regimens can be suggested. Eventually, such tools could be used for individual patients with complicated infectious disease histories or as personalized antibiograms in ambulatory settings. This might benefit those with frequent infections such as UTI, dialysis patients with repeated sepsis, or cystic fibrosis patients with highly specific ecologies.

Our data underscore the potential benefits of more specific antibiograms as well as the continued need for careful, case-based consideration of therapeutic options by expert clinicians. Although the data presented here support the utility of multidimensional antibiograms, they also highlight the challenges in designing a balanced tool that offers generalizability to multiple syndromes and presents therapeutic options that account for the immediate need for broad coverage and the counterpoised threat of antibiotic resistance in the clinical environment. Combining associated mortality data that is specific to certain patient populations may help navigate this risk so that narrow-spectrum antibiotic regimens may be used in low-risk scenarios and broader spectrum treatments could be saved for short periods in high-stakes scenarios. Table 1 illustrates this point. Here, we see that iBiogram not only distinguished between syndromes with no mortality (UTI) and those with high (sepsis), similar to providers, but tailored treatments further based on other risk factors. Notably, providers prescribed 74% (48/65) to 75.9% (364/479) of watch antibiotics in UTI, whereas iBiogram recommended watch group therapies only in UTI cases with prior antibiotic use. In sepsis, 91% (21/23) to 95% (71/75) of provider prescriptions were the watch group and 2.1% (8/383) to 4% (3/75) reserve antibiotics, whereas the iBiogram system suggested reserve antibiotics only in high mortality cases, providing better empiric coverage.

### Limitations and Future Directions

The retrospective nature of the study and the quality of the EHR data are the two primary limitations. Retrospectively distinguishing pathogenic organisms from contaminants can be challenging. Therefore, we excluded many positive cultures that did not have a corresponding ICD-10 code. Moreover, many infections are treated without culture or negative cultures are ignored. This leads to a selection bias in the analyzed EHR data, whereby only positive cultures are recorded, likely resulting in a bias toward higher acuity diseases. The presented factors rely mainly on ICD-10 codes that (1) limit granularity, (2) are prone to coding errors, and (3) may not reflect important uncharted factors, including severity and timing of disease. For instance, it was not possible to distinguish sepsis from severe sepsis based on coding alone and medical data such as blood pressure, or use of pressers, were beyond the scope of this work. Furthermore, not all antibiotics were tested against all the isolates. For example, levofloxacin is rarely tested at our institution because its resistance patterns closely match those of ciprofloxacin. Finally, new antibiotics are continuously brought to the market to treat resistant organisms, and retrospective data will, by definition, not include the newest available antimicrobials.

Similar to traditional antibiograms, the prototype described in this work currently focuses only on antibiotic coverage and does not consider other factors such as side effects, or restricted institutional antibiotics, nor does it distinguish the mode of delivery (intravenous vs oral) and the cost or risk of interactions with other medications. It also did not present patient-specific data with regard to recent specific antibiotic exposure or past personal culture data. Finally, the current version of the iBiogram did not include data on newer broad-spectrum antimicrobials such as ceftolozane-tazobactam and ceftazidime-avibactam, which may offer more reasonable and less toxic options in the setting of a CRE or CRPA.

Finally, we propose that these methods may be applied to inter–health care system data to create regional antibiograms [36-38] or with international databases such as WHONET data, where the platform could also extend to resource-limited and rural settings [39]. Therefore, data sets grow in breadth, depth, and history, machine learning tools and recursive approaches may increasingly be applied to predict the drivers of ecology and patterns of resistance.

### Conclusions

Overall, we aimed to create an interactive shared representation of the complex factors that clinicians must navigate for effective empiric prescriptions. To this end, we developed a CDSS that strives to promote effective collaboration with users, bridging the gap in presentation and reasoning between clinical guidelines, clinicians’ expertise, and data science by representing local outcomes and local ecology in a user-centered tool. Overall, we propose that such tools could improve clinical and safety outcomes, reduce adverse events because of inappropriate antibiotics, provide real-time suggestions to prescribers, and lower AMR and costs of care.

## Appendix

Multimedia Appendix 1 Definitions and International Classification of Diseases-10 (ICD-10) mapping. Overview of definitions used for factors, including the mapping of ICD-10 values to syndromes and comorbidities.

Multimedia Appendix 2 Overview of cohort and culture properties after removing duplicate cultures showing the number of patients, visits, isolates, and susceptibility tests for each factor.

Multimedia Appendix 3 Infection ecology over time demonstrates the ecology of infection-causing bacteria in the hospital system. Printed antibiograms generally consider only the past quarter of the hospital data. Here, we analyze the past quarter’s ecology compared with the past two quarters, past three quarters, etc. Overlaid represents the *P* value of each comparison. As shown, the bacterial ecology when considering the past four quarters is statistically similar (*P*>.05) to that of the past quarter, suggesting that we can use up to four quarters).

Multimedia Appendix 4 All factors influencing microbial prevalence (left) and resistance (right). Odds ratios for statistically significant factors (*P*<.05) influencing the probability of encountering a specific bacterium or an isolate resistant to a particular treatment were adjusted by sex and age. Sex and age were adjusted for each other. Factors included demographic information, zip code, medications, syndromes, and comorbidities as coded in the International Classification of Diseases-10. In the left graph, purple indicates a higher prevalence of pathogens. In the right graph, red indicates a higher and blue indicates a lower prevalence of resistant isolates.

Multimedia Appendix 5 Patient home address influencing microbial prevalence (left) and resistance (right). Odds ratios for statistically significant factors (*P*<.05) influencing the probability of encountering a specific bacterium or an isolate resistant to a particular treatment were adjusted by sex and age. Factors included demographic information, zip code, medications, syndromes, and comorbidities as coded in the ICD-10. In the left graph, purple indicates a higher prevalence of pathogens. In the right graph, red indicates a higher and blue indicates a lower prevalence of resistant isolates.

Multimedia Appendix 6 Factors influencing microbial prevalence (left) and resistance (right) in sepsis Odds ratios for statistically significant factors (*P*<.05) influencing the probability of encountering a specific bacterium or an isolate resistant to a particular treatment were adjusted by sex and age. Factors included demographic information, zip code, medications, syndromes, and comorbidities as coded in the International Classification of Diseases-10. In the left graph, purple indicates a higher prevalence of pathogens. In the right graph, red indicates a higher and blue indicates a lower prevalence of resistant isolates.

## Abbreviations

AMR: antimicrobial resistance
CDSS: clinical decision support system
CRE: carbapenem-resistant Enterobacteriaceae
CRPA: carbapenem-resistant Pseudomonas aeruginosa
EHR: electronic health record
ICD-10: International Classification of Diseases-10
LOS: length of stay
OR: odds ratio
UCSDHS: University of California San Diego Health Sciences
UTI: urinary tract infection
