# Supplementary material for: A Risk-Based Clinical Decision Support System for Patient-Specific Antimicrobial Therapy (iBiogram): Design and Retrospective Analysis
Source: J Med Internet Res. 2021 Dec 3;23(12):e23571. doi: 10.2196/23571 (PMC8686485; doi:10.2196/23571)
Supplement: Multimedia Appendix 1 [file jmir_v23i12e23571_app1.doc]

***Supplemental Table 1 Definitions and*** ***ICD Mapping****. Overview of definitions used for factors including the mapping of ICD-10 values to syndromes and comorbidities.*

| Term | Definition |
| --- | --- |
| CRE | Enterobacteriaceae that test resistant to at least one of the carbapenem antibiotics |
| Inpatient | Culture obtained while patient was admitted to an inpatient ward |
| ICU | Culture obtained while patient was in an Intensive Care Unit |
| ED | Culture obtained while patient was in an Emergency Department |
| Healthcare Facility-Onset | Isolates were obtained at least 3 days after an inpatient was admitted |
| Immunosuppressed | Isolates were obtained after immunosuppressants were prescribed to the patient.  The immunosuppressants recorded in the dataset follow:  Corticosteroids  prednisone (Deltasone, Orasone)  budesonide (Entocort EC)  prednisolone (Millipred)  Janus kinase inhibitors  tofacitinib (Xeljanz)  Calcineurin inhibitors  cyclosporine (Neoral, Sandimmune, SangCya)  tacrolimus (Astagraf XL, Envarsus XR, Prograf)  mTOR inhibitors  sirolimus (Rapamune)  everolimus (Afinitor, Zortress)  IMDH inhibitors  azathioprine (Azasan, Imuran)  leflunomide (Arava)  mycophenolate (CellCept, Myfortic)  Biologics  abatacept (Orencia)  adalimumab (Humira)  anakinra (Kineret)  certolizumab (Cimzia)  etanercept (Enbrel)  golimumab (Simponi)  infliximab (Remicade)  ixekizumab (Taltz)  natalizumab (Tysabri)  rituximab (Rituxan)  secukinumab (Cosentyx)  tocilizumab (Actemra)  ustekinumab (Stelara)  vedolizumab (Entyvio)  Monoclonal antibodies  basiliximab (Simulect)  daclizumab (Zinbryta) |
| Prior Antibiotics | Isolates were obtained between 1-30 days after antibiotics were prescribed to the patient  153 Antibiotics are recorded in the dataset |
| Patient Location (e.g. Central San Diego | Defined according to patient ZIP code |
| **Syndromes**: Inferred from ICD-10 Codes at time of order and discharge | |
| UTI | Positive urine culture and ICD 10 Codes within:  N39.0 Urinary tract infection, site not specified  N30* Cystitis, unspecified without hematuria |
| Sepsis (including Bacteremia) | Positive blood culture and ICD Codes within:  A02.1* (Salmonella sepsis)  A32.7* (Listerial sepsis)  A40* (Streptococcal sepsis)  A41* (Sepsis)  A47.7* (Actinomycotic sepsis)  B37.7* (Candidal sepsis)  O85* (Puerperal sepsis)  P36* (Bacterial sepsis of newborn)  R65.20 (Severe sepsis without septic shock)  R65.21 (Severe sepsis with septic shock) |
| Bacteremia | Positive blood culture and no sepsis code |
| Pneumonia | Positive respiratory culture and ICD Codes within:  J09.X1 (Influenza due to identified novel influenza A virus with pneumonia)  J10.0* (Influenza due to other identified influenza virus with unspecified type of pneumonia)  J10.08  J11.0* (Influenza due to unidentified influenza virus with pneumonia)  J12* (Viral pneumonia)  J13* (Pneumonia due to Streptococcus pneumoniae)  J14* (Pneumonia due to Hemophilus influenzae)  J15* (Bacterial pneumonia)  J16* (Pneumonia due to other infectious organisms)  J17* (Pneumonia in diseases classified elsewhere)  J18* (Pneumonia, unspecified organism)  J84.111(Idiopathic interstitial pneumonia)  J84.116 (Cryptogenic organizing pneumonia)  J84.2 (Lymphoid interstitial pneumonia)  J85.1* (Abscess of lung with pneumonia)  J95.851 (Ventilator associated pneumonia)  P23* (Congenital pneumonia  Z87.01 (Recurrent pneumonia) |
| Hospital Acquired Pneumonia (HAP) | Patient has pneumonia with at least one isolate obtained a minimum of 3 days after admission |
| Community Acquired Pneumonia (CAP) | Patient has pneumonia with isolates obtained before or less than 3 days after admission |
| Skin and Soft Tissue Infections (SSTI) | ICD-10 Codes within:  E11.621 (Type 2 diabetes mellitus with foot ulcer)  H00* (Hordeolum and chalazion)  H05.01* (Cellulitis of orbit)  H60.0* (Abscess of external ear)  H60.10 (Cellulitis of external ear, unspecified ear)  J34.0* (Abscess, furuncle and carbuncle of nose)  K12.2 (Cellulitis and abscess of mouth)  K61* (Abscess of anal and rectal regions)  L01* (Impetigo)  L02* (Cutaneous abscess, furuncle and carbuncle)  L03* (Cellulitis and acute lymphangitis)  L08* (Other local infections of skin and subcutaneous tissue)  M71* (Other bursopathies)  M72.6 (Necrotizing fasciitis)  N48.21 (Abscess of corpus cavernosum and penis)  N48.22 (Cellulitis of corpus cavernosum and penis)  N61.1 (Abscess of the breast and nipple)  N73.0 (Acute parametritis and pelvic cellulitis)  N73.1 (Chronic parametritis and pelvic cellulitis)  N73.2 (Unspecified parametritis and pelvic cellulitis)  N76.4* (Abscess of vulva)  AND positive Skin/Soft Tissue cultures |
| Post-Surgical Complications | ICD-10 Codes within:  E89* (Postprocedural endocrine and metabolic complications and disorders)  G89.18 (Other acute postprocedural pain)  G89.28 (Other chronic postprocedural pain)  G97* (Intraoperative and postprocedural complications and disorders of nervous system)  H59* (Intraoperative and postprocedural complications and disorders of eye and adnexa)  I95.81 (Postprocedural hypotension)  I97* (Intraoperative and postprocedural complications and disorders of circulatory system)  J95* (Intraoperative and postprocedural complications and disorders of respiratory system)  K66.0 (Peritoneal adhesions (postprocedural) (postinfection))  K68.11 (Postprocedural retroperitoneal abscess)  K91* (Intraoperative and postprocedural complications and disorders of digestive system)  L76* (Intraoperative and postprocedural complications of skin and subcutaneous tissue)  M96* (Intraoperative and postprocedural complications and disorders of musculoskeletal system, not elsewhere classified)  N99* (Intraoperative and postprocedural complications and disorders of genitourinary system)  T81* (Complications of procedures)  T82* (Complications of cardiac and vascular prosthetic devices, implants and grafts)  T83* (Complications of genitourinary prosthetic devices, implants and grafts)  T84* (Complications of internal orthopedic prosthetic devices, implants and grafts)  T85* (Complications of other internal prosthetic devices, implants and grafts)  T86* (Complications of transplanted organs and tissue)  T87* (Complications peculiar to reattachment and amputation)  Z48* (Encounter for other postprocedural aftercare)  Z95.8 (Presence of other cardiac and vascular implants and grafts)  Z98.890 (Other specified postprocedural states) |
| osteomyelitis | ICD Codes within:  H05.02* (Osteomyelitis of orbit)  M46.2* (Osteomyelitis of vertebra)  M86* (Osteomyelitis) |
| endocarditis | ICD-10 Codes within:  A39.51 (Meningococcal endocarditis)  A52.03 (Syphilitic endocarditis)  B37.6 (Candidal endocarditis)  I01.1 (Acute rheumatic endocarditis)  I33.0 (Acute and subacute infective endocarditis)  I33.9  (Acute and subacute endocarditis, unspecified)  I38 (Endocarditis, valve unspecified)  I39 (Endocarditis and heart valve disorders in diseases classified elsewhere) |
| fever | ICD-10 Codes within:  A01 (Typhoid and paratyphoid fevers)  A68* (Relapsing fevers)  A78* (Q fever)  A92* (Other mosquito-borne viral fevers)  A99 (Unspecified viral hemorrhagic fever  I00* (Rheumatic fever without heart involvement)  L98.2 (Febrile neutrophilic dermatosis)  R50* (Fever)  R56.0* (Febrile convulsions) |
| Neutropenic Fever | Patient has fever  AND  another ICD-10 Code within:  D70.3 (Neutropenia due to infection)  D70.8 (Other neutropenia)  D70.9 (Neutropenia, unspecified)  P61.5 (Transient neonatal neutropenia) |
| **Comorbidities**: Inferred from ICD10 Codes in problem list, at order, and before order | |
| Diabetes | ICD-10 Codes within:  E08* (Diabetes mellitus due to underlying condition)  E09* (Drug or chemical induced diabetes mellitus)  E10* (Type 1 diabetes mellitus)  E11* (Type 2 diabetes mellitus)  E13* (Other specified diabetes mellitus)  E23.2 (Diabetes insipidus)  N25.1 (Nephrogenic diabetes insipidus) |
| HIV | ICD-10 Codes within:  B20* (Human immunodeficiency virus [HIV] disease)  B97.35 (Human immunodeficiency virus, type 2 [HIV 2] as the cause of diseases classified elsewhere)  O98.7* (Human immunodeficiency virus [HIV] disease complicating pregnancy, childbirth and the puerperium)  Z11.4  Z20.6 (Contact with and (suspected) exposure to human immunodeficiency virus [HIV])  Z21* (Asymptomatic human immunodeficiency virus [HIV] infection status) |
| Bone Marrow Transplant | ICD-10 Codes within:  T86.0* (Complications of bone marrow transplant)  T86.5* (Complications of stem cell transplant)  Z48.290 (Encounter for aftercare following bone marrow transplant)  Z94.81 (Bone marrow transplant status)  Z94.84 (Stem cell transplant status) |
| Solid Tumor | ICD-10 Codes C00 - C80 inclusively |
| Hematologic Malignancy | ICD-10 Codes C81 - C96 |
| Hypertension | ICD-10 Codes within:  I10* (Essential (primary) hypertension)  I15* (Secondary hypertension)  I87.3* (Chronic venous hypertension (idiopathic))  O10* (Pre-existing hypertension complicating pregnancy, childbirth and the puerperium)  O11* (Pre-existing hypertension with pre-eclampsia) |
| Acute Renal Failure | ICD-10 Codes within:  N17* (Acute kidney failure) |
| Chronic Kidney Disease (CKD) | ICD-10 Codes within:  D63.1 (Anemia in chronic kidney disease)  E08.22 (Diabetes mellitus due to underlying condition with diabetic chronic kidney disease)  E10.22 (Type 1 diabetes mellitus with diabetic chronic kidney disease)  E11.22 (Type 2 diabetes mellitus with diabetic chronic kidney disease)  E13.22 (Other specified diabetes mellitus with diabetic chronic kidney disease)  I12* (Hypertensive chronic kidney disease)  I13* (Hypertensive heart and chronic kidney disease)  N18* (Chronic kidney disease (CKD))  O10.213 (Pre-existing hypertensive chronic kidney disease complicating pregnancy, third trimester) |
| Heart Failure | ICD-10 Codes within:  I11.0 (Hypertensive heart disease with heart failure)  I13.0 (Hypertensive heart and chronic kidney disease with heart failure and stage 1 through stage 4 chronic kidney disease, or unspecified chronic kidney disease)  I13.2 (Hypertensive heart and chronic kidney disease with heart failure and with stage 5 chronic kidney disease, or end-stage renal disease)  I50* (Heart failure) |
| Urinary Catheter | ICD-10 Codes within:  T83.01* (Breakdown (mechanical) of urinary catheter)  T83.02* (Displacement of urinary catheter)  T83.09* (Other mechanical complication of urinary catheter)  T83.1* (Mechanical complication of other urinary devices and implants)  T83.51* (Infection and inflammatory reaction due to urinary catheter)  Z46.6 (Encounter for fitting and adjustment of urinary device)  Z96.0 (Presence of urogenital implants) |
| Vascular Catheter | ICD-10 Codes within:  T80.21* (Infection due to central venous catheter)  T82.4* (Mechanical complication of vascular dialysis catheter)  Z45.2 (Encounter for adjustment and management of vascular access device) |
| Hemodialysis | ICD-10 Codes within:  I95.3 (Hypotension of hemodialysis)  T82.4* (Mechanical complication of vascular dialysis catheter)  Z49.01 (Encounter for fitting and adjustment of extracorporeal dialysis catheter)  Z99.2 (Dependence on renal dialysis) |
| Depression | ICD-10 Codes within:  F06.31 (Mood disorder due to known physiological condition with depressive features)  F25.1 (Schizoaffective disorder, depressive type)  F32* (Major depressive disorder, single episode)  F33* (Major depressive disorder, recurrent) |
| Lung Transplant | ICD-10 Codes within:  T86.81* (Complications of lung transplant)  Z48.2* (Encounter for aftercare following organ transplant)  Z94.2 (Lung transplant status)  Z94.3 (Heart and lungs transplant status) |
| Liver Transplant | ICD-10 Codes within:  T86.4* (Complications of liver transplant)  Z48.23 (Encounter for aftercare following liver transplant)  Z94.4 (Liver transplant status) |
| Kidney transplant | ICD-10 Codes within:  T86.1 (Complications of kidney transplant)  Z48.22 (Encounter for aftercare following kidney transplant)  Z94.0 (Kidney transplant status) |
| Heart Transplant | ICD-10 Codes within:  I25.811 (Atherosclerosis of native coronary artery of transplanted heart without angina pectoris)  I25.812 (Atherosclerosis of bypass graft of coronary artery of transplanted heart without angina pectoris)  T86.2* (Complications of heart transplant)  Z94.1 (Heart transplant status)  Z94.3 (Heart and lungs transplant status) |
| Cystic Fibrosis | ICD-10 Codes within:  E84* (Cystic fibrosis) |
| Deceased | Provided by hospital system along with date |
