# Supplementary material for: A Risk-Based Clinical Decision Support System for Patient-Specific Antimicrobial Therapy (iBiogram): Design and Retrospective Analysis
Source: J Med Internet Res. 2021 Dec 3;23(12):e23571. doi: 10.2196/23571 (PMC8686485; doi:10.2196/23571)
Supplement: Multimedia Appendix 2 [file jmir_v23i12e23571_app2.doc]

***Supplemental Table 2*** ***Dataset Summary****. Overview of cohort and culture properties after removing duplicate cultures showing the number of patients, visits, isolates and susceptibility tests for each factor.*

|  |  | **Patients** | **Encounters** | **Isolates** | **Susceptibility Tests** |
| --- | --- | --- | --- | --- | --- |
|  | **Count** | 30716 | 55770 | 74021 | 831399 |
| **Demographics** | **Mean Age (Min, Max)** | 54.6 (1, 103) | 55.6 (1, 103) | 55.3 (1, 103) | 56.0 (1, 103) |
|  | **Sex (m)** | 11823 (38.5%) | 21017 (37.7%) | 31392 (42.4%) | 310850 (37.4%) |
|  | **White** | 18577 (60.5%) | 35180 (63.1%) | 45730 (61.8%) | 515869 (62.0%) |
|  | **Other Race or Mixed Race** | 6161 (20.1%) | 10802 (19.4%) | 15081 (20.4%) | 166438 (20.0%) |
|  | **Black or African American** | 2234 (7.3%) | 4018 (7.2%) | 5689 (7.7%) | 61721 (7.4%) |
|  | **Asian** | 2441 (7.9%) | 3903 (7.0%) | 4908 (6.6%) | 59007 (7.1%) |
| **Encounter** | **Outpatient** |  | 25995 (46.6%) | 29845 (40.3%) | 367578 (44.2%) |
|  | **ED** |  | 7260 (13.0%) | 8061 (10.9%) | 104469 (12.6%) |
|  | **Inpatient** |  | 22515 (40.4%) | 36115 (48.8%) | 359352 (43.2%) |
|  | **Hospital Facility-onset** | | 4275 (7.7%) | 9821 (13.3%) | 87750 (10.6%) |
|  | **ICU** |  | 1177 (2.1%) | 3103 (4.2%) | 26390 (3.2%) |
| **Risk Factors and Comorbidities** | **Prior Antibiotic use** | | 16046 (28.8%) | 24166 (32.6%) | 237780 (28.6%) |
|  | **On Immunosuppression** | | 4028 (7.2%) | 6641 (9.0%) | 61848 (7.4%) |
|  | **Resident in Correctional Facility** | | 142 (0.3%) | 200 (0.3%) | 1894 (0.2%) |
|  | **Active IV Drug Use** | | 166 (0.3%) | 233 (0.3%) | 2324 (0.3%) |
|  | **Urinary Catheter** | | 2299 (4.1%) | 3338 (4.5%) | 38201 (4.6%) |
|  | **Vascular Catheter** | | 5285 (9.5%) | 10784 (14.6%) | 96471 (11.6%) |
|  | **Diabetes** |  | 14702 (26.4%) | 20431 (27.6%) | 223029 (26.8%) |
|  | **Cystic Fibrosis** |  | 2938 (5.3%) | 4462 (6.0%) | 39492 (4.8%) |
|  | **ESRD on Hemodialysis** | | 1728 (3.1%) | 3183 (4.3%) | 30019 (3.6%) |
|  | **HIV** |  | 2266 (4.1%) | 2932 (4.0%) | 30745 (3.7%) |
|  | **Lung Transplant** | | 798 (1.4%) | 1351 (1.8%) | 11100 (1.3%) |
|  | **Kidney Transplant** | | 1327 (2.4%) | 1758 (2.4%) | 19227 (2.3%) |
|  | **Liver Transplant** | | 640 (1.1%) | 1033 (1.4%) | 9998 (1.2%) |
|  | **Heart Transplant** | | 210 (0.4%) | 398 (0.5%) | 3763 (0.5%) |
|  | **Solid Tumor** |  | 12358 (22.2%) | 16621 (22.5%) | 186581 (22.4%) |
|  | **Hematologic Malignancy** | | 2454 (4.4%) | 3346 (4.5%) | 34506 (4.2%) |
|  | **Bone Marrow Transplant** | | 956 (1.7%) | 1327 (1.8%) | 12430 (1.5%) |
|  | **Acute Renal Failure** | | 11394 (20.4%) | 18141 (24.5%) | 182936 (22.0%) |
|  | **CKD** |  | 8168 (14.6%) | 11669 (15.8%) | 126216 (15.2%) |
|  | **COPD** |  | 3587 (6.4%) | 4908 (6.6%) | 52000 (6.3%) |
|  | **Cirrhosis** |  | 2565 (4.6%) | 3845 (5.2%) | 39084 (4.7%) |
|  | **Hypertension** |  | 22613 (40.5%) | 30099 (40.7%) | 341104 (41.0%) |
|  | **Heart Failure** |  | 6057 (10.9%) | 8823 (11.9%) | 94857 (11.4%) |
| **Syndromes** | **UTI** |  | 15892 (28.5%) | 18416 (24.9%) | 247662 (29.8%) |
|  | **Bacteremia** |  | 2114 (3.8%) | 3059 (4.1%) | 22527 (2.7%) |
|  | **Sepsis** |  | 1852 (3.3%) | 3252 (4.4%) | 25600 (3.1%) |
|  | **Skin/Soft Tissue Infection** | | 2194 (3.9%) | 3199 (4.3%) | 31158 (3.7%) |
|  | **Osteomyelitis** |  | 1092 (2.0%) | 2548 (3.4%) | 22160 (2.7%) |
|  | **Complicated CAP** | | 1669 (3.0%) | 2649 (3.6%) | 24677 (3.0%) |
|  | **HAP/VAP** |  | 698 (1.3%) | 2283 (3.1%) | 18783 (2.3%) |
|  | **Endocarditis** |  | 458 (0.8%) | 1250 (1.7%) | 8700 (1.0%) |
|  | **Neutropenic Fever** | | 452 (0.8%) | 848 (1.1%) | 6655 (0.8%) |
|  | **Post-Surgical Complications** | | 3413 (6.1%) | 6844 (9.2%) | 62450 (7.5%) |
| **Sample** | **Urine** |  |  | 37669 (50.9%) | 506281 (60.9%) |
|  | **Respiratory** |  |  | 12698 (17.2%) | 115909 (13.9%) |
|  | **Skin/Soft Tissue** | |  | 6987 (9.4%) | 66429 (8.0%) |
|  | **Blood** |  |  | 6311 (8.5%) | 48127 (5.8%) |
|  | **Gram Positive** |  |  | 22098 (29.9%) | 167518 (20.1%) |
