# Supplementary figures and images for: A Risk-Based Clinical Decision Support System for Patient-Specific Antimicrobial Therapy (iBiogram): Design and Retrospective Analysis
Source: J Med Internet Res. 2021 Dec 3;23(12):e23571. doi: 10.2196/23571 (PMC8686485; doi:10.2196/23571)

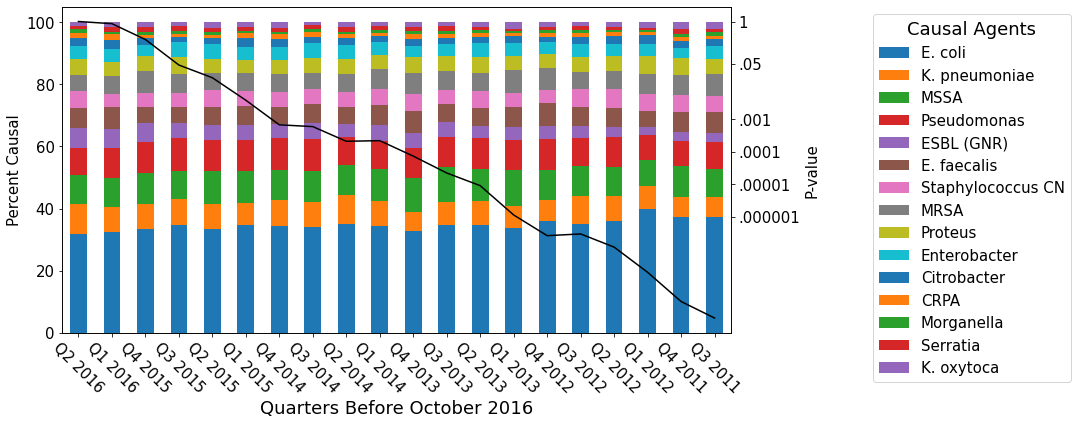

Supplement: Multimedia Appendix 3 [file jmir_v23i12e23571_app3.png]

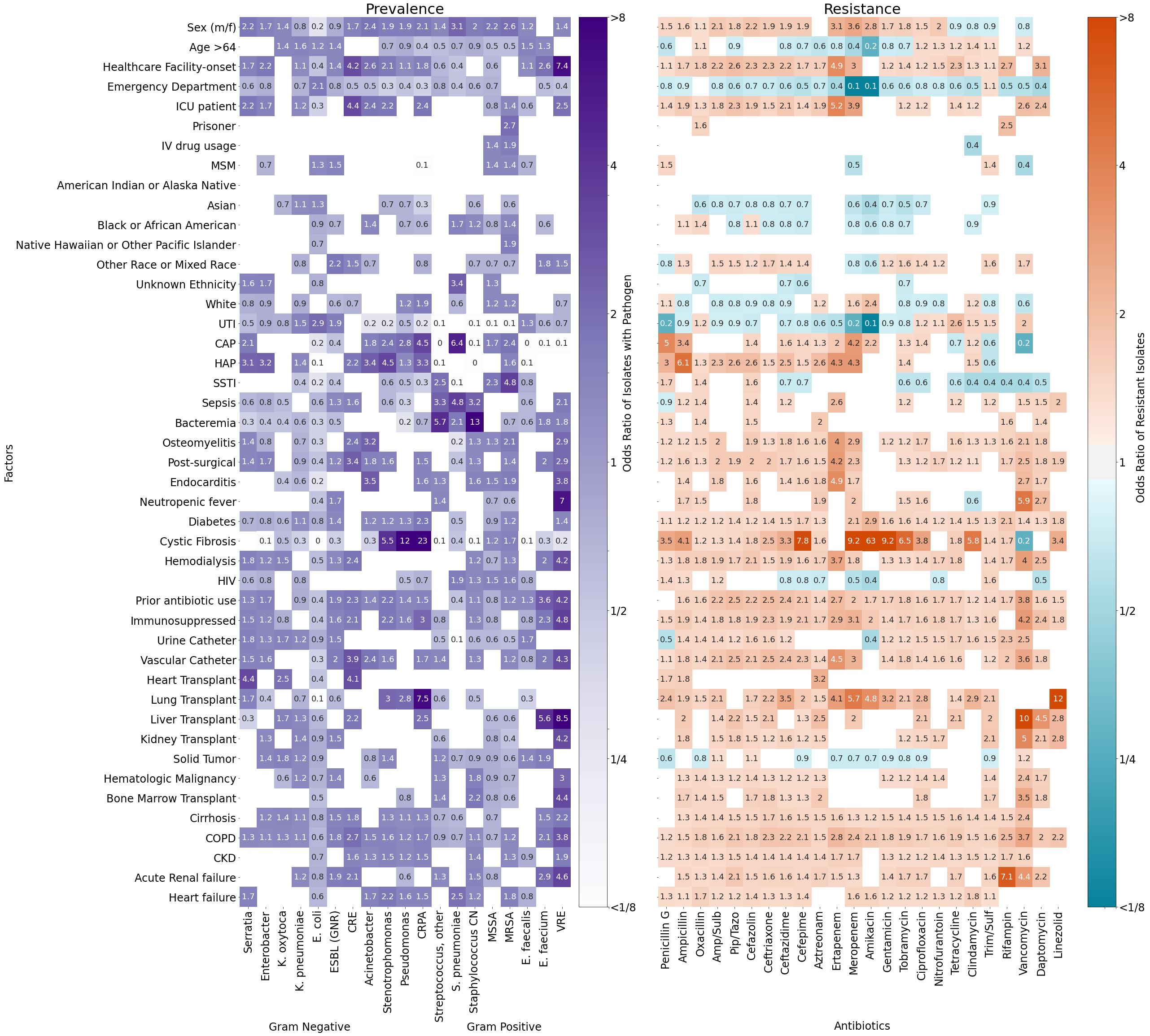

Supplement: Multimedia Appendix 4 [file jmir_v23i12e23571_app4.png]

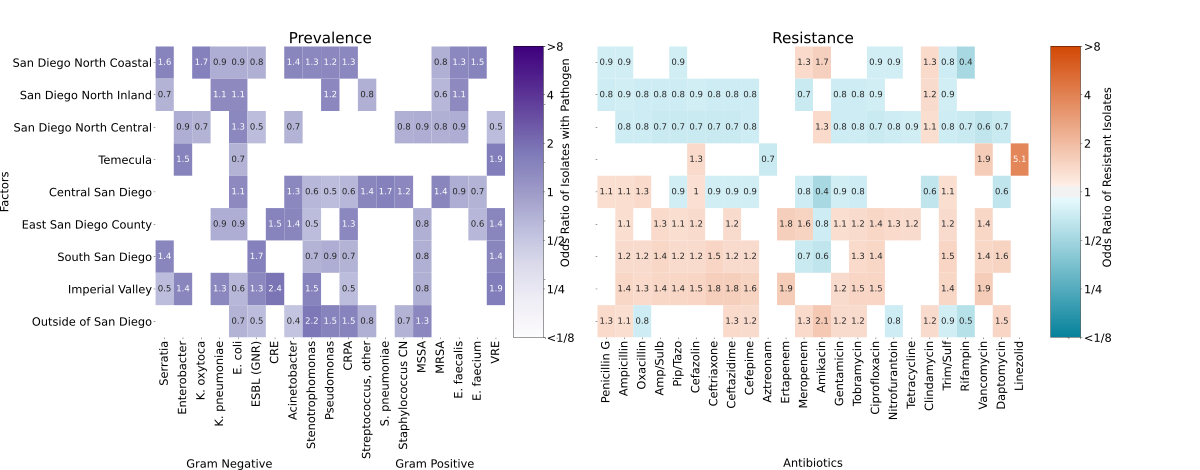

Supplement: Multimedia Appendix 5 [file jmir_v23i12e23571_app5.png]

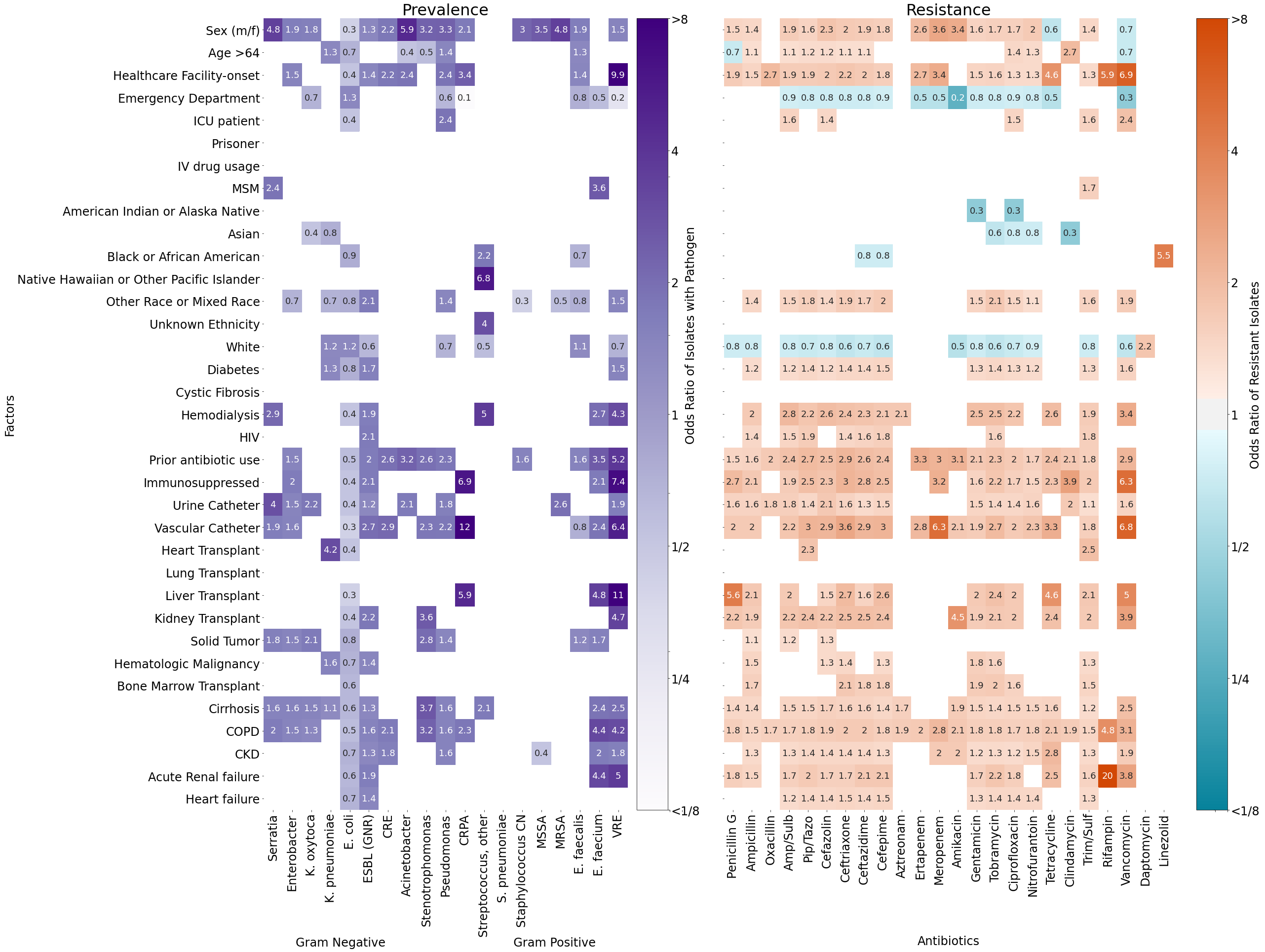

Supplement: Multimedia Appendix 6 [file jmir_v23i12e23571_app6.png]
